# Integrating Infectious Disease Advanced Practice Providers in the Workforce: An Educational Step Forward

**DOI:** 10.1093/ofid/ofae232

**Published:** 2024-04-30

**Authors:** Leah H Yoke, Michael Boeckh, Alison M Beieler

**Affiliations:** Division of Allergy and Infectious Diseases, University of Washington, Seattle, Washington, USA; Vaccine and Infectious Disease Division, Fred Hutchinson Cancer Center, Seattle, Washington, USA; Division of Allergy and Infectious Diseases, University of Washington, Seattle, Washington, USA; Vaccine and Infectious Disease Division, Fred Hutchinson Cancer Center, Seattle, Washington, USA; Infectious Diseases Clinic, Harborview Medical Center, Seattle, Washington, USA

**Keywords:** advanced practice providers, infectious disease education, infectious disease workforce, nurse practitioners, physician assistants

Advanced practice providers (APPs) are an expanding clinical workforce and are being used in a wide variety of medical specialties to provide increased access to quality care. In the specialty of infectious diseases (ID), there is less use of these skilled clinicians, and little is understood about the role they broadly play within the ID medical team when they are used. Given the physician shortages that plague ID, this clinical workforce might provide an opportunity to reimagine the ID workforce of the future.

In a recent issue of *Open Forum Infectious Diseases,* Smith et al describe clinical competencies and curriculum development formulated for training APPs in ID that aligns with 6 core competencies of the Accreditation Council for Graduate Medical Education and the American Board of Medical Specialties [1]. These 6 core competencies include patient care, medical knowledge, practice-based learning and environment, interpersonal and communication skills, professionalism, and system-based practices. Within these larger competencies, they proposed milestones of achievement at 3-, 6-, and 12-month windows. They also described general principles of infectious disease with competencies and objectives for achievement by the APP, with the ultimate goal of onboarding and training additional APPs in ID.

We believe this paper is important as the current ID workforce continues to be stretched and APPs could be a valuable member of the ID team of the future, increasing quality care and providing additional access. Across the United States, there is uneven distribution of ID physicians, and using APPs as physician-extenders in underserved areas could offer enhanced care to these populations [2]. Although groups including MD Anderson, University of Pittsburgh Medical Center, and Lurie Children's Hospital have described integration of APPs into ID, little has been published concerning education and onboarding [3, 4, 5]. The paper by Smith et al gives a blueprint and a methodology for this, with milestones of achievement and ID knowledge [1]. However, to fully optimize the use of APPs, more educational resources are needed for both onboarding new APPs and continuing clinical education [6]. In fact, we know of no other outline of educational training and competencies for ID APPs currently in publication.

Other specialties have taken strategic steps to provide these kinds of resources for APPs and for medical groups considering APPs. In 2015, The American College of Rheumatology noted similar shortages in their workforce. The Association of Rheumatology Professions and Rheumatology Research Foundation developed specific resources and learning modules for APPs to aid practices in onboarding and incorporating APPs [7]. Fiscal resources were also offered to offset any lost revenues while onboarding and training APPs [8]. All these strategic interventions allowed for additional APPs to be recruited and trained into this specialty. Other medical specialties including GI and oncology have also created robust training plans for APPs and successfully integrated them into practices nationwide [9, 10]. Further, a recent publication at an academic medical center suggested interprofessional collaboration with physicians and development of structured support for APPs to practice at the top of their license, which is allowed for improved access across multiple specialties [11].

The Infectious Diseases Society of America (IDSA) maintains its mission to “improve the health of individuals, communities, and society by promoting excellence in patient care, education, research, public health, and prevention relating to infectious diseases” [12]. We believe interprofessional collaboration between ID-trained physicians, and other nonphysician members of the care team will be crucial to continue forward progress of this mission. Although there is an ongoing need for increasing numbers of board-certified ID physicians to enable success of the future ID team, expansion of the ID workforce to include nonphysician colleagues will also be key as the national medical landscape continues to evolve. With that evolution and expansion comes the urgent need for educational resources, as outlined in the paper by Smith et al.

IDSA has recently developed a new workforce plan that specifically calls for the creation of educational and training resources for APPs, with corresponding strategies from a business perspective to justify the hiring and training of APPs [13]. The paper by Smith et al is therefore timely and provides a practical and important blueprint to foster the ID team of the future. However, the proposed curriculum is extensive, and the proposed timelines and expectations are ambitious. To successfully implement the proposed curriculum and to achieve the long-needed and strategic step to foster inclusion of APPs into the ID team, more work and resources are needed. These include the development of educational resources, designated training courses, core competencies and performance metrics for onboarding followed by continued professional development and training for these important members of the medical team. Also needed are evaluations of these metrics and usable data to understand the optimal way to educate APPs and use them in our medical teams. A curriculum task force as part of the new IDSA workforce could help achieve these goals. Securing fiscal resources to offset any lost revenues while onboarding and training APPs as this paper describes is also crucial [8].

Access to quality ID care may become more challenging as the health care landscape changes. Recognizing APPs as a clinician workforce that can extend ID care to meet the growing population is vital. Strategic steps taken together in partnership with ID physicians to provide high-quality education and training for APPs must be a priority for the entire ID community.

## Notes

***Patient consent statement.*** This paper does not include factors necessitating patient consent.
